# Epidemiological characteristics and survival analysis on patients with occupational pneumoconiosis in Zhejiang Province from 1987 to 2019

**DOI:** 10.3389/fpubh.2022.1006391

**Published:** 2022-10-14

**Authors:** Hua Zou, Zhihao Shi, Yixin Zhang, Jiena Zhou, Xinglin Fang, Yijin Zhang, Yong Hu, Xiaoming Lou, Lifang Zhou

**Affiliations:** ^1^Occupational Health and Radiation Protection Institute, Zhejiang Center for Disease Control and Prevention, Hangzhou, China; ^2^Jiaxing Center for Disease Control and Prevention, Jiaxing, China; ^3^School of Medicine, Hangzhou Normal University, Hangzhou, China; ^4^Department of Public Health, School of Medicine, Zhejiang University, Hangzhou, China; ^5^School of Public Health, Xiamen University, Xiamen, China

**Keywords:** occupational disease, pneumoconiosis, dust-exposure, survival analysis, Kaplan-Meier

## Abstract

**Objective:**

To evaluate risk factors affecting survival in patients diagnosed with pneumoconiosis and propose strategies to improve the quality of life in these patients.

**Methods:**

The basic patient information was obtained from the pneumoconiosis report card. Disease types, regions, and industry distribution of pneumoconiosis were analyzed. The Kaplan-Meier survival curves and the Cox proportional risk regression model was used for survival analysis.

**Results:**

A total of 13,812 patients were diagnosed with pneumoconiosis in Zhejiang province from 1987 to 2019. The overall survival rate at the end of life table analysis was 83%. Kaplan-Meier analyses showed that there were significant differences between survival curves depending on the stage of first diagnosis, age at first diagnosis, type of pneumoconiosis, industry, and duration of dust exposure (*P* < 0.05). The results of Cox proportional hazards regression analysis showed that pneumoconiosis stage of first diagnosis, age at first diagnosis, industry, and duration of dust exposure were risk factors affecting patient survival (*P* < 0.05).

**Conclusions:**

The patients with high stage of pneumoconiosis at first diagnosis, older age, and long duration of dust exposure should be followed up and monitored as key population, and the industries with high incidence of pneumoconiosis such as mining and construction should be supervised as key industries.

## Introduction

Pneumoconiosis is the most widely distributed occupational disease worldwide. Pneumoconiosis is characterized by diffuse fibrosis of the lung tissue that is caused by long-term inhalation and retention of productive mineral dust in the lungs during occupational activities. According to the Global Burden of Disease Study 2017, the number of confirmed cases of pneumoconiosis was increased from 36,186 cases in 1990 to 60,055 in 2017 (1). In 2017, silicosis was the most common type of pneumoconiosis worldwide, followed by coal worker pneumoconiosis (CWP). In recent decades, with the rapid economic development, accelerated urban construction, and increased demand for mineral resources in China, a large number of enterprises with backward production technology, incomplete protective equipment, and inadequate health management have emerged in small mines and small smelting industries. Meanwhile, with the increase in migrant workers, the probability of people who are employed in related industries in China to suffer from pneumoconiosis has increased. There are ~26,000–28,000 newly reported cases of pneumoconiosis in China every year (2). According to the Report on Occupational Disease Prevention and Control issued by the Ministry of Health of China from 2010 to 2018, pneumoconiosis ranks first in incidence among occupational diseases every year, accounting for more than 70% of the cases (3), making it the leading occupational disease in China.

The main clinical manifestations of pneumoconiosis include dyspnea, cough, expectoration, and chest pain. Currently, there is no effective treatment for lung injury caused by pneumoconiosis (4). Even if the patients are no longer exposed to the dust, lung function damage gradually worsens as the disease progresses, and complications such as tuberculosis, corpulmonale, and chronic obstructive pulmonary disease (COPD) may occur. Currently, the main treatments include symptomatic and rehabilitation treatments. Patients are being followed up and reexamined regularly, and the treatment plan is adjusted according to the patient's condition (5). In addition, patients are advised to strengthen nutrition, perform physical exercise, and maintain a healthy lifestyle to alleviate symptoms, delay disease progression, prolong survival time, and reduce the incidence of complications. As a lifelong occupational disease, pneumoconiosis shortens the survival time in patients, reduces their ability to work, and causes great loss to their health and economy.

To prevent pneumoconiosis, the International Labor Organization (ILO) and the World Health Organization (WHO) jointly launched a global pneumoconiosis elimination plan, aiming at eradicating pneumoconiosis worldwide by 2030. In 2016, China issued the National Occupational Disease Control Program (2016–2020) to promote the construction of healthy China and further protect laborer rights and interests. The governance of occupational pneumoconiosis is a key task in this program (6). This study aimed to evaluate the incidence of pneumoconiosis and its influencing factors in the context of the living conditions in patients in Zhejiang Province. Further, this study aimed to develop strategies to prevent the occurrence and development of pneumoconiosis, reduce associated complications, prolong the survival, and improve the quality of life in patients. To this end, the newly reported occupational pneumoconiosis patients in Zhejiang Province from 1987 to 2019 were followed up. The epidemiological characteristics of pneumoconiosis and patient survival were analyzed, with the aim to provide a scientific basis for the prevention and treatment of pneumoconiosis in Zhejiang Province.

## Materials and methods

### Study population

Patients who were diagnosed with pneumoconiosis according to the Diagnosis of Occupational Pneumoconiosis (GBZ70-2015) should be reported in the national occupational disease report card by occupational disease diagnosis agencies promptly (7). The national occupational disease report card contained the general information (e.g., name, sex, age, job, industry, and years exposed to dust) and disease information (e.g., date of diagnosis, and the type and stage of pneumoconiosis). Through the report card, patients diagnosed with pneumoconiosis in Zhejiang Province from 1987 to 2019 were included in this study. The survival situation of these patients was obtained from the police system which took charge of census register. The insurance, e.g., basic medical insurance, employment injury insurance, and employer compensation, these patients had was obtained from social security departments, medical security departments, civil affairs departments, and human resources departments. The patients' underlying causes of death were obtained through the residential cause of death surveillance system. All information was checked by telephone follow-up. The study protocol was approved by the ethics committee of the Zhejiang Center for Disease Control and Prevention, China (approval reference number: 2022-026-01).

All cases were divided into two categories: death cases and censored cases. The following classification criteria were used: death cases corresponded to the patients who died of pneumoconiosis and its complications (such as chronic obstructive pulmonary disease, tuberculosis, emphysema, etc.); censored cases corresponded to either those who survived after follow-up, or died of other causes unrelated to pneumoconiosis (such as accidents, diabetes, leukemia, etc.) during the follow-up period, or were lost to follow-up. The survival time in patients with pneumoconiosis in this study was defined as the time period between the year of first diagnosis and year of death.

### Diagnosis of occupational pneumoconiosis

According to the pneumoconiosis diagnostic criteria in China—Diagnosis of Occupational Pneumoconiosis (GBZ70-2015), experts in occupational disease diagnosis should make the diagnosis after taking many elements into consideration such as the occupational history exposed to mineral dust, clinical manifestation, posterior-anterior chest X-ray radiographs, epidemiological survey data on pneumoconiosis, the working environment, etc. The small opacity and pleural plague were the typical characteristics of X-ray image of pneumoconiosis. Comparing to a diagnostic standard radiograph for pneumoconiosis, the conclusion could be drawn about whether occupational pneumoconiosis was present and the type and stage of pneumoconiosis. It could be grouped into three stages based on the X-ray image: Stage I, Stage II, and Stage III. The increase in grade represented the progression of pneumoconiosis. Patients could progress from Stage I to Stage II, from Stage II to Stage III, or directly from Stage I to Stage III.

### Statistical analysis

Excel 2016 was used to build the patient database. Descriptive and survival analyses were performed using SPSS 21.0. Continuous variables were presented as mean with standard deviation, and categorical variables were presented as proportions. Analysis of variance was used to compare continuous variables between different groups and chi-square test was used for categorical variables. The Kaplan-Meier survival curves were plotted and the log-rank test was used to compare the survival curves of different groups. With outcome and survival times being the dependent variables and the potential risk factors being the independent variables, the univariate Cox regression model was conducted firstly for screening, and then the multivariate Cox regression model was conducted to analyze the influencing factors for patient survival. The *P* < 0.05 was considered significant.

## Results

### Epidemiological characteristics

#### General information

**Figure 1:**
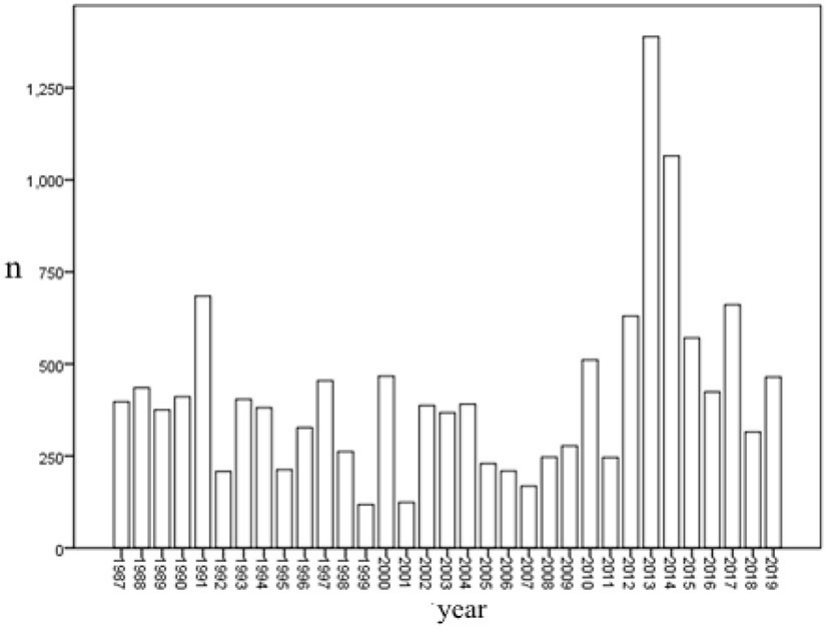
Pneumoconiosis incidence in Zhejiang Province from 1987 to 2019.

A total of 13,812 patients were diagnosed with pneumoconiosis in Zhejiang province from 1987 to 2019. Among them, 96.7% (13,356) were men and 3.3% (456) women. As shown in Figure 1, the number of confirmed pneumoconiosis cases in 2013 and 2014 was higher than that in other years. Among all the confirmed pneumoconiosis cases, there were 8,437 cases (61.1%) in Stage I, 2,738 cases (19.8%) in Stage II, and 2,637 cases (19.1%) in Stage III. Table 1 shows that silicosis was the most common type (8,896 cases, 64.4%) among all types of pneumoconiosis, followed by CWP (2,835 cases, 20.5%).

**Table 1:** Stage distribution of different types of pneumoconiosis cases in Zhejiang Province from 1987 to 2019.

**Types of pneumoconiosis**	**Stage of pneumoconiosis**			**Total**
	**I**	**II**	**III**	
Silicosis	4,838	1,779	2,279	8,896
CWP	1,873	701	261	2,835
Graphite pneumoconiosis	22	4	1	27
Carbon black pneumoconiosis	11	3	1	15
Asbestosis	110	14	1	125
Talc pneumoconiosis	5	5	2	12
Cement pneumoconiosis	658	33	8	699
Mica pneumoconiosis	1	4	0	5
Pottery worker's pneumoconiosis	39	6	6	51
Aluminosis	14	7	0	21
Welder's pneumoconiosis	273	22	3	298
Founder pneumoconiosis	78	5	4	87
Others	515	155	71	741
Total	8,437	2,738	2,637	13,812

CWP, coal worker pneumoconiosis.

#### Progressed stages of pneumoconiosis

There were totally 894 progressed cases of pneumoconiosis from 1987 to 2019. There were statistically significant differences in the average period of pneumoconiosis progression between progression from Stage I to Stage II, progression from Stage II to Stage III, and progression from Stage I to Stage III (*F* = 7.141, *P* = 0.001). It took an average of (7.01 ± 6.34) years for pneumoconiosis patients at Stage I to progress to Stage II, which was not statistically different (*P* > 0.05) from the average period (7.84 ± 7.14 years) of pneumoconiosis progression from Stage II to Stage III. There was also no significant difference in the average period of pneumoconiosis progression between progression from Stage II to Stage III and progression from Stage I to Stage III. As a contrast, it took much longer for pneumoconiosis patients to progress from I to III than it did to progress from I to II (*t* = −3.719, *P* < 0.001). The progression of pneumoconiosis stages among the different types is shown in Table 2. For the progression from I to II and from II to III, there was no significant difference between the types of pneumoconiosis (*P* > 0.05). For the progression from I to III, there was a significant difference between different types of pneumoconiosis (*F* = 11.352, *P* < 0.001).

**Table 2:** Disease progression between stages among patients with different types of pneumoconiosis in Zhejiang Province from 1987 to 2019.

**Stage of pneumoconiosis**	***n***	**years**	**Silicosis**	**CWP**	**Others**	***F***	***P***
I–II	534	7.01 ± 6.34	7.33 ± 5.94	6.79 ± 7.13	6.14 ± 5.57	1.035	0.356
II–III	158	7.84 ± 7.14	7.35 ± 6.69	9.81 ± 8.82	7.00 ± 4.06	1.555	0.214
I–III	202	9.04 ± 6.74	7.83 ± 5.85	13.05 ± 7.99	10.86 ± 7.62	11.352	<0.001

CWP, coal worker pneumoconiosis.

#### Regional distribution

The top three cities reporting the most cases of pneumoconiosis from 1987 to 2019 were Quzhou (3,048 cases, 22.5%), Taizhou (3,038 cases, 21.5%), and Huzhou (1,706 cases, 16.4%), accounting for 56.4% of all pneumoconiosis cases in Zhejiang. In addition, in Quzhou and Huzhou, the CWP cases were most frequently reported among all cases, while silicosis cases were most frequently reported in other regions (Table 3).

**Table 3:** Regional distribution of pneumoconiosis cases reported in Zhejiang Province from 1987 to 2019.

**Cities**	**Silicosis (*n*)**	**CWP (*n*)**	**Others (*n*)**	**Total**
Hangzhou	1,233	196	128	1,557
Ningbo	828	3	512	1,343
Wenzhou	1,300	11	86	1,397
Jiaxing	127	5	73	205
Huzhou	713	843	150	1,706
Shaoxing	190	0	59	249
Jinhua	502	47	101	650
Quzhou	562	1,708	778	3,048
Zhoushan	112	1	46	159
Taizhou	2,969	7	62	3,038
Lishui	360	14	86	460
Total	8,896	2,835	2,081	13,812

CWP, coal worker pneumoconiosis.

#### Industry distribution

From 1987 to 2019, the non-metallic mineral processing industry (3,972 cases, 28.76%), coal mining (2,503 cases, 18.13%), public administration, social security, and social organization (1,632 cases, 11.81%), non-metallic mineral products industry (1,561 cases, 11.30%), and construction industry (770 cases, 5.57%) ranked the top five among all industries, accounting for 75.2% of the total pneumoconiosis cases. A total of 6,814 people were confirmed in the coal mining industry, accounting for 49.3% of the total pneumoconiosis cases (Table 4).

**Table 4:** Industry distribution of pneumoconiosis cases reported in Zhejiang Province from 1987 to 2019.

**Industry type**	**Silicosis (*n*)**	**CWP (*n*)**	**Other (*n*)**	**Total**
Mining				
Coal mining and washing	392	2,055	56	2,503
Non-metallic mining	3,695	169	63	3,927
Other mining industries	363	12	9	384
Manufacturing				
Manufacture of non–metallic mineral product	659	122	780	1,561
Other manufacturing industries	1,340	195	729	2,264
Construction	603	9	158	770
Public administration, social organization and social security	1,283	222	127	1,632
Other industries	561	51	159	771
Total	8,896	2,835	2,081	13,812

The pneumoconiosis at Stage I had the highest constituent ratio in all industries. In the mining industry, Stage I, II and III accounted for 60.3% (3,678 cases), 19.1% (1,493 cases) and 20.6% (1,643 cases) of total pneumoconiosis cases, respectively (Table 5).

**Table 5:** Industry distribution of pneumoconiosis of each stage at first diagnosis in Zhejiang Province from 1987 to 2019.

**Industry type**	**I (*n*)**	**II (*n*)**	**III (*n*)**	**Total**
Mining				
Coal mining and washing	1,620	621	262	2,503
Non-metallic mining	1,821	770	1,336	3,927
Other mining industries	237	102	45	384
Manufacturing				
Manufacture of non–metallic mineral product	1,282	176	103	1,561
Other manufacturing industries	1,676	371	217	2,264
Construction	546	157	67	770
Public administration, social organization and social security	775	362	495	1,632
Other industries	480	179	112	771
Total	8,437	2,738	2,637	13,812

#### Dust-exposed working years

Table 6 shows the dust-exposed working years for different types of pneumoconiosis. From 1987 to 2019, the average dust-exposed working years among pneumoconiosis patients in Zhejiang was (13.9 ± 8.6) years, the median exposure time was 12.6 years, and the longest dust-exposed working years was 45.3 years. Pottery worker's pneumoconiosis had the longest mean years of dust exposure (17.5 ± 9.3 years), and mica pneumoconiosis had the shortest mean dust exposure (9.6 ± 0.9 years). Talc pneumoconiosis had the shortest median exposure to dust (i.e., 6.8 years). There were significant differences in years of dust exposure between the different types of pneumoconiosis (*F* = 12.276, *P* < 0.001). Among all confirmed cases, those with dust-exposed working years between 5 and 10 years had the highest rate (21.1%), while there were only 87 cases (0.6%) with dust-exposed working years ≥40.0, as shown in Table 7.

**Table 6:** Dust-exposed working years for different types of pneumoconiosis in Zhejiang Province from 1987 to 2019.

**Type of pneumoconiosis**	**Dust-exposed working years**		
	***n***	**Mean** ±**SD**	**Median**
Silicosis	8,896	14.2 ± 8.7	13
CWP	2,835	13.5 ± 8.2	13
Graphite pneumoconiosis	27	15.0 ± 8.8	12.8
Carbon black pneumoconiosis	15	12.6 ± 8.0	9
Asbestosis	125	14.8 ± 7.3	13.2
Talc pneumoconiosis	12	9.7 ± 7.0	6.8
Cement pneumoconiosis	699	11.1 ± 7.8	10.2
Mica pneumoconiosis	5	9.6 ± 0.9	9.5
Pottery worker's pneumoconiosis	51	17.5 ± 9.3	18.6
Aluminosis	21	9.9 ± 5.5	11
Welder's pneumoconiosis	298	11.4 ± 7.2	9.7
Founder pneumoconiosis	87	16.6 ± 9.5	15
Other	713	14.2 ± 9.2	11.4
Total	13,812	13.9 ± 8.6	12.6

CWP, coal worker pneumoconiosis.

**Table 7:** Dust-exposed working years of confirmed pneumoconiosis cases in Zhejiang Province from 1987 to 2019.

**Dust-exposed working years**	***n***	**%**
<5.0	2,332	16.9
5.0~	2,912	21.1
10.0~	2,676	19.4
15.0~	2,643	19.1
20.0~	1,680	12.2
25.0~	780	5.6
30.0~	533	3.9
35.0~	169	1.2
≥40.0	87	0.6
Total	13,812	100

#### Age distribution

As shown in Table 8, the average age at first diagnosis of these patients was 52.4 years, and the median age was 52.0 years. At first diagnosis, the patients with various types of pneumoconiosis were mostly in the 40- and the 50-year-old age groups, accounting for 27.8 and 33.9%, respectively. The average age of patients first diagnosised with Stage I, II, and III pneumoconiosis was (51.0 ± 11.3), (54.5 ± 10.6), and (55.8 ± 8.9) years, respectively. There was a statistically significant difference in the age at first diagnosis of pneumoconiosis patients at different stages (*F* = 241.78, *P* < 0.001).

**Table 8:** Age distribution at first diagnosis in patients with various types of pneumoconiosis in Zhejiang Province from 1987 to 2019.

**Age(year)**	**Silicosis**		**CWP**		**Others**		**Total**	
	***n***	**%**	***n***	**%**	***n***	**%**	***n***	**%**
<30	148	1.7	39	1.4	71	3.4	258	1.9
30	687	7.7	289	10.2	450	21.6	1,426	10.3
40	2,129	23.9	958	33.8	749	36	3,836	27.8
50	3,290	37	913	32.2	483	23.2	4,686	33.9
60	2,014	22.6	514	18.1	252	12.1	2,780	20.1
70	562	6.3	112	4	68	3.3	742	5.4
≥80	66	0.7	10	0.4	8	0.4	84	0.6
Total	8,896	100	2,835	100	2,081	100	13,812	100

CWP, coal worker pneumoconiosis.

#### Medical insurance for the confirmed cases

Among the patients, 93.2% had basic medical insurance, 58.7% had serious disease insurance, and 139 (1.4%) patients had no insurance (Table 9). There was no statistically significant difference in the medical insurance coverage in patients with pneumoconiosis at different stages (χ^2^ = 2.523, *P* = 0.283), but there were significant differences in the coverage of employment injury insurance (χ^2^ = 289.524, *P* < 0.001) and employer compensation (χ^2^ = 150.080, *P* < 0.001) in patients with pneumoconiosis at different stages (Table 10).

**Table 9:** Insurance for prevalent cases of occupational pneumoconiosis.

**Type of insurance**	**Pneumoconiosis**	**Covered by**	
	**patients**	**insurance**	
		***n***	**%**
Employment injury insurance	9,754	3,037	31.1
Employer compensation		1,669	17.1
Basic medical insurance		9,089	93.2
Serious disease insurance		5,722	58.7
Other		1,869	19.2
No insurance		139	1.4

**Table 10:** Insurance for prevalent cases of occupational pneumoconiosis at various stages.

**Insurance**	**Stage of pneumoconiosis**			**χ^2^**	***P***
	**I**	**II**	**III**		
Basic medical insurance					
Yes	5,572	1,738	1,179		
No	429	152	84	2.523	0.283
Employment injury insurance				289.524	<0.001
Yes	2,203	533	301		
No	3,798	1,357	1,563		
Employer compensation				150.08	<0.001
Yes	1,198	327	144		
No	4,803	1,563	1,719		

### Survival and influencing factors analyses

#### Survival analysis

Among the 13,812 confirmed pneumoconiosis patients in Zhejiang Province, 9,754 were still alive, 3,285 died, and 773 were lost to follow-up. The average age at death was 68.1 years (range, 26.0–100.0). A total of 873 patients died of pneumoconiosis and associated complications, accounting for 26.6% of the deaths, with an average course of 11.0 years.

**Figure 2:**
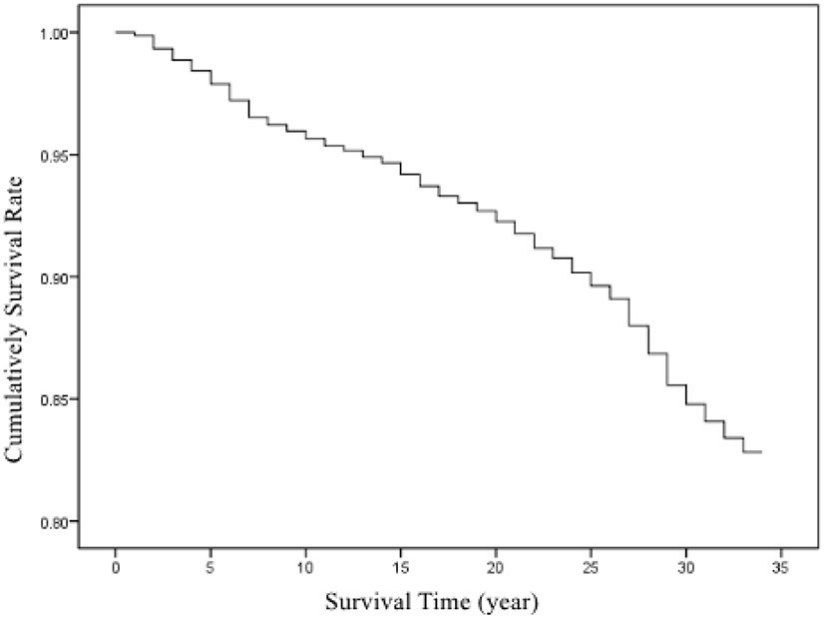
Cumulative survival curves in patients with confirmed cases of pneumoconiosis in Zhejiang Province from 1987 to 2019.

Among the patients with occupational pneumoconiosis from 1987 to 2019, the shortest survival time was < 1 year, and the longest 33 years. The survival rates in patients with occupational pneumoconiosis 10, 20, and 30 years after diagnosis were 95, 92, and 84%, respectively. Finally, the cumulative survival rate was 83%. The cumulative survival curves in patients are shown in Figure 2.

#### Analysis of factors influencing survival in pneumoconiosis patients

##### Impact of pneumoconiosis stage at first diagnosis on the survival time

**Figure 3:**
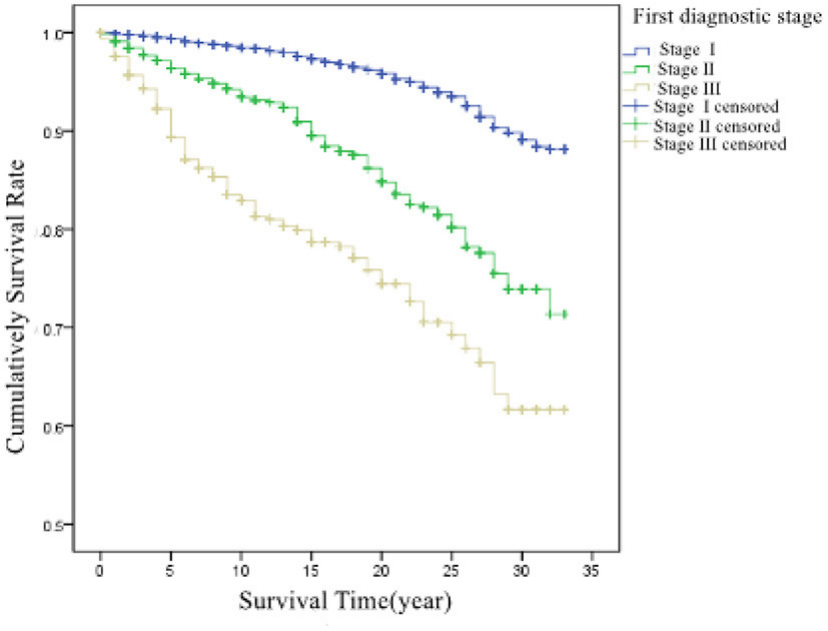
Cumulative survival rate in patients at different stages at first diagnosis.

According to the pneumoconiosis stage at first diagnosis, patients were divided into three groups: stage I, II, and III, with an average survival time of 31.6, 28.9, and 25.8 years, respectively. There was a significant difference in survival between the different stages at the first diagnosis (χ^2^ = 863.673, *P* < 0.001). The survival curves of the patients are shown in Figure 3.

##### Impact of age at first diagnosis on survival time

**Figure 4:**
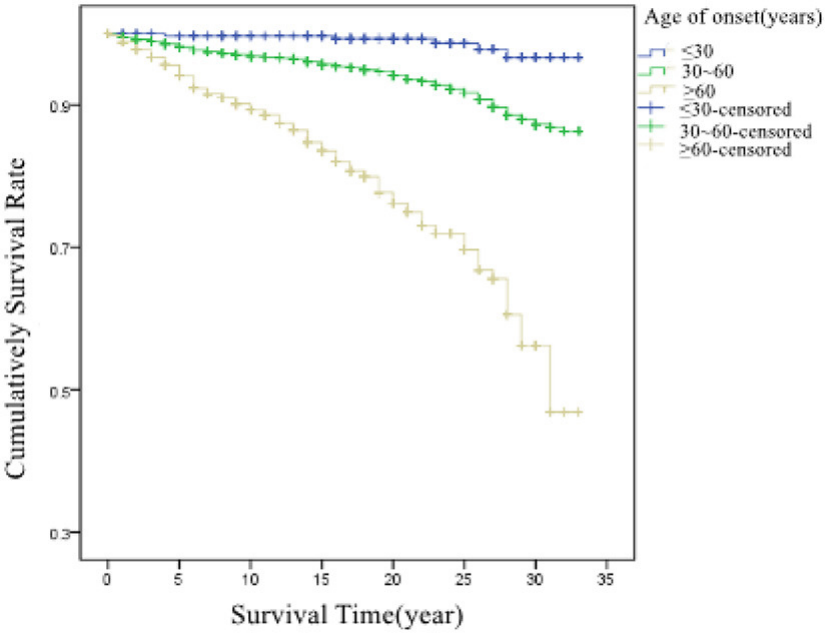
Cumulative survival rate in patients at different ages at first diagnosis.

Based on the age at first diagnosis of pneumoconiosis, patients were divided into three groups: ≤30 years old, 31–60 years old, and ≥61 years old, and their average survival time were 32.7 years, 31.2 years, and 26.2 years, respectively. There was a significant difference in survival curves between the different age groups (χ^2^ = 414.302, *P* < 0.001). The survival curves of the patients are shown in Figure 4.

##### Impact of industry on the survival time

**Figure 5:**
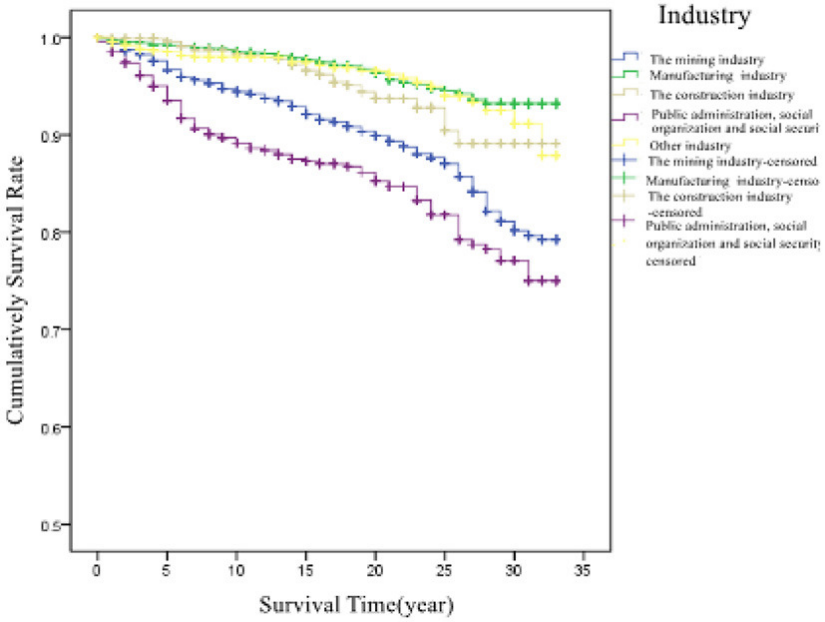
Cumulative survival rate in patients in different industry.

According to the industry, patients were divided into five groups: mining, manufacturing, construction, public administration and social organization and social security, and other industries. Their average survival times in these industries were 29.9, 32.0, 31.4, 28.6, and 31.8 years, respectively. There was a significant difference in survival time between the different industries (χ^2^ = 57.266, *P* < 0.001).The corresponding survival curves of the patients are shown in Figure 5.

##### Impact of pneumoconiosis types on the survival time

**Figure 6:**
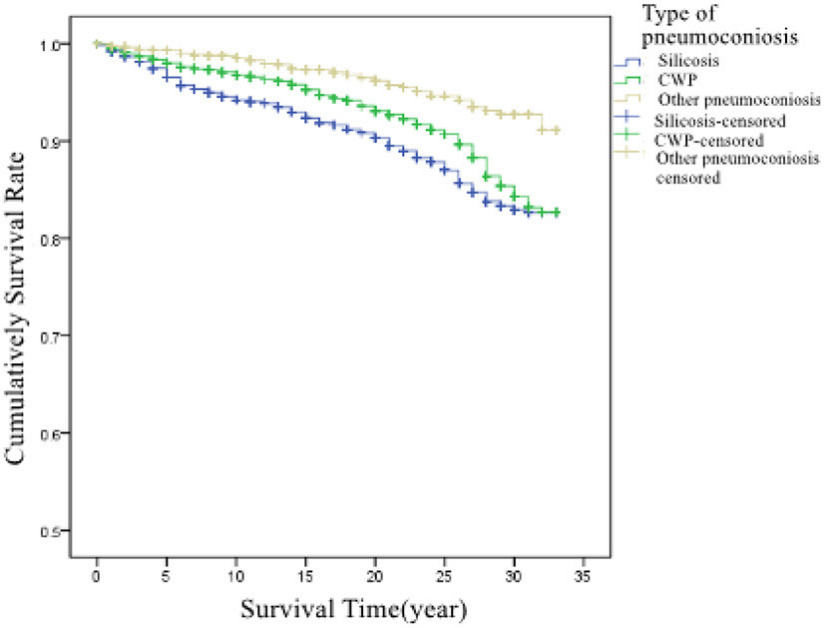
Cumulative survival rate in patients with different pneumoconiosis types.

Patients were divided into three groups according to the type of pneumoconiosis: silicosis, CWP, and other pneumoconiosis. Their corresponding average survival times were 30.1, 30.9, and 31.9 years, respectively. There were statistically significant differences in survival times between the groups with different pneumoconiosis types (χ^2^= 76.229, *P* < 0.001). The survival curves of the patients are shown in Figure 6.

##### Impact of dust-exposed working years on the survival time

**Figure 7:**
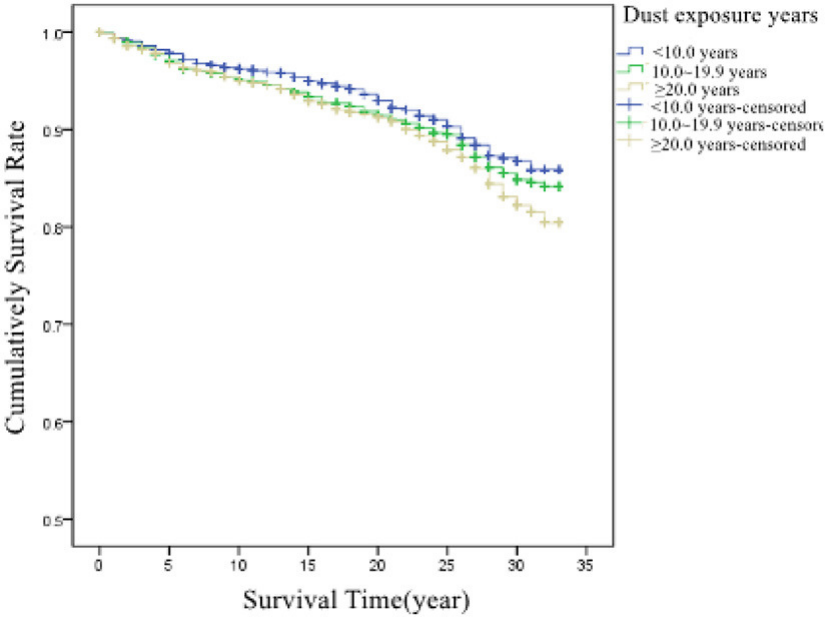
Cumulative survival rate in patients with different years of dust exposure.

According to the dust-exposed working years, patients were divided into three groups: <10.0, 10.0–19.9, and ≥20.0 years. The average survival times in these three groups were 30.9, 30.5, and 30.3 years, respectively. There were statistically significant differences in survival times between the groups with different years of dust exposure (χ^2^= 10.288, *P* = 0.006). The survival curves of the patients are shown in Figure 7.

#### The Cox regression analyses

Table 11 shows the results of Cox regression analyse. Based on the available data, there were six potential risk factors influencing the survival in patients including sex, pneumoconiosis stage at first diagnosis, age at first diagnosis, industry, type of pneumoconiosis, and dust-exposed working years. The univariate Cox regression analyses between patient survival and each factor showed that there was no significant difference of the effect on patient survival between males and females (*P* = 0.351). The multivariate Cox regression analysis for the remaining five factors showed that pneumoconiosis stage at first diagnosis, age at first diagnosis, industry, and dust-exposed working years were the risk factors influencing the survival time of pneumoconiosis patients, while type of pneumoconiosis was not statistically significant in this model (*P* > 0.05). Patients with higher stage at first diagnosis, older age at first diagnosis, longer duration of dust exposure, or from mining and public administration and social organization and social security industries had higher hazard ratio of death.

**Table 11:** The Cox regression analysis.

**Variables**	**Groups**	**Univariate Cox regression**				**Multivariate Cox regression**			
		***β***	***P***	**Exp (*β*)**	**95.0% *CI***	***β***	***P***	**Exp (*β*)**	**95.0% *CI***
Sex	Male^*^								
	Female	−0.189	0.351	0.828	0.556–1.232				
Stage at first diagnosis	Stage I^*^								
	Stage II	1.201	<0.001	3.322	2.785–3.964	1.034	<0.001	2.813	2.347–3.370
	Stage III	2.136	<0.001	8.465	7.196–9.959	1.908	<0.001	6.741	5.656–8.034
Age at first diagnosis	≤30 years^*^								
	~60 years	1.630	<0.001	5.105	2.116–12.314	1.549	0.001	4.708	1.948–11.377
	>60 years	2.980	<0.001	19.694	8.124–47.740	2.710	<0.001	15.025	6.177–36.551
Industry	Others^*^								
	Mining	0.908	<0.001	2.479	1.706–3.603	0.965	<0.001	2.624	1.794–3.839
	Manufacturing	−0.196	0.347	0.822	0.547–1.236	0.431	0.041	1.539	1.017–2.328
	Construction	0.219	0.387	1.245	0.758–2.044	0.562	0.027	1.755	1.067–2.888
	Public administration, social organization and social security	1.416	<0.001	4.121	2.779–6.110	1.349	<0.001	3.853	2.592–5.728
Type of pneumoconiosis	Others ^*^								
	Silicosis	1.007	<0.001	2.736	2.146–3.490	0.211	0.117	1.234	0.948–1.607
	CWP	0.697	<0.001	2.007	1.542–2.611	0.207	0.167	1.230	0.917–1.649
Dust-exposure years	<10.0 years^*^								
	<20.0 years	0.184	0.021	1.202	1.028–1.405	0.140	0.088	1.150	0.980–1.350
	≥20 years	0.271	0.002	1.311	1.103–1.559	0.231	0.010	1.260	1.056–1.504

* Reference group; CI, confidence interval; CWP, coal worker pneumoconiosis.

## Discussion

Presently, pneumoconiosis is the leading occupational disease in China. There is no effective cure for this condition, which means it is a lifelong disease. Pneumoconiosis seriously endangers the health of the occupational population and poses a great burden on society. Studies have shown that due to differences in enterprise types and scales, protective measures, government supervision, health education, and so on, the epidemiological characteristics of pneumoconiosis in different regions are also different.

The type of pneumoconiosis in Zhejiang Province was mainly silicosis, followed by CWP. This was different from the distribution of the main pneumoconiosis types in Guangdong Province (8), Hunan Province, Xinjiang Uygur Autonomous Region, and Tianjin. Silicosis ranked first in Guangdong Province, followed by welder pneumoconiosis and other pneumoconiosis. CWP was the leading type of pneumoconiosis in Hunan Province and Xinjiang Uygur Autonomous Region, followed by silicosis. Silicosis and caster pneumoconiosis were the main types of pneumoconiosis in Tianjin. These could be attributed to the local industrial structure and distribution of natural resources. There were a large number of shipbuilding enterprises in Guangdong Province, leading to many cases of welder's pneumoconiosis. Hunan and Xinjiang were main distribution areas of coal mines in China, and CWP was the leading pneumoconiosis type there. Tianjin was a city with developed processing industries and had no mining resources, as a result of which CWP was not the main type of pneumoconiosis. In Zhejiang Province, there were coal resources dominated by non-ferrous and non-metallic mines, therefore silicosis had the largest number of cases. Coal resources in Zhejiang were mainly distributed in Quzhou and Huzhou, which agreed with the results of this study. The results showed that most silicosis cases came from industries that produced silicium dust during production and operation, such as non-metallic mineral processing, non-metallic mineral products, and construction industries. Furthermore, 72.5% of CWP cases came from the coal mining and washing industry. These results agree with the findings of a national study that silicosis cases were common in the railway industry, building materials industry, non-ferrous metal industry, and metallurgy industry, and CWP was common in the coal mining and washing industry (9).

This study found that the highest number of new cases was in 2013 and 2014. The possible reasons include: (1) According to the relevant requirements of the Notice on Standardized Construction of Occupational Disease Diagnosis and Identification in Zhejiang Province [(2012) No.93] formulated by the Health Department of Zhejiang Province, in the second half of 2012, cities and counties began to allocate more staff and equipment for occupational disease diagnosis and strengthen personnel training, carry out publicity and training in hospitals, and actively cultivate third-party intermediaries with detecting qualifications; (3) In 2013, the Ministry of Health formulated Administrative Measures for Diagnosis and Identification of Occupational Diseases (Order No.91 of the Ministry of Health). In 2014, the Zhejiang Provincial Health and Health Commission revised and improved the original regulations and formulated the Working Regulations for Occupational Disease Identification in Zhejiang Province, which was distributed to all cities and counties. Similar observations were reported by other studies in which the results were affected by these policies (10).

This study found that patients working in the mining industry had a worse survival than those working in other industries. The proportions of stage II and stage III pneumoconiosis at first diagnosis in the mining industry were higher than those in other industries, which may have been due to the fact that dust exposure in the mining industry was more than in other industries and led to faster progression of the disease (11). In addition, “public administration, social security and social organizations,” non-metallic mineral products industry, and construction industry were associated with a high number of confirmed pneumoconiosis cases. This may be attributed to the fact that patients used to work in dust-exposed posts in their early careers. A number of mining enterprises have been successively shut down in the past 30 years in Zhejiang Province. Due to the long incubation period of pneumoconiosis, workers were uniformly organized by the government for health examination after the enterprises were shut down or went bankrupt, and workers diagnosed with pneumoconiosis were recorded and reported. The results suggest that the local government should pay attention to the prevention and control of occupational diseases and strengthen the supervision and management of enterprises.

The progression from stage I to stage III took longer than that from stage I to stage II. Factors affecting the degree of dust exposure and type of dust, such as dust exposure duration, type of work, and personal protection, could influence the time of progression. Generally, the more dust exposure, the faster the disease progresses, and the shorter the time of progression. This study found that it took shorter for silicosis to progress from stage I to stage III than CWP and other types of pneumoconiosis. This may be attributed to the higher content of free silica and more harm to health for silicious dust than other dusts. Relevant studies also showed that the survival in silicosis patients was worse than that in patients with other types of pneumoconiosis (12, 13).

The results of Cox regression analyses in this study showed that dust-exposure working years was an important risk factor affecting the survival time in patients, which was similar to the results of other studies (14). It is reported that dust exposure could affect the death of patients (15), which may be because the long-term employment in the dust-exposed industries increases the cumulative dust exposure of the workers. Related studies have also shown that even dust with relatively small damage to humans could cause great loss to the health in patients when cumulative dust exposure is large (16). It is necessary to carry out regular physical examinations to detect pneumoconiosis as early as possible. In addition, the results also showed that from 1987 to 2019, 16.9% of these cases were diagnosed with pneumoconiosis only after a short-term exposure to dust for <5 years, demonstrating the need to strengthen the protection for dust-exposed workers in Zhejiang Province.

There are still some deficiencies in the system construction and coverage of social insurance for occupational disease patients in China, which leads to the failure to implement protection for some patients (17). Among the confirmed cases in Zhejiang Province, although most people were covered by basic medical insurance, the coverage of employment injury insurance and employer compensation was still not comprehensive, let alone the fact that there were 139 patients without any type of insurance. This implied that the coverage was not comprehensive enough, which indirectly aggravated the difficulty in pneumoconiosis prevention and control (18). Therefore, occupational disease management needs to be further strengthened. Our results showed that there were statistically significant differences in the coverage rate of employment injury insurance and employer compensation between stage I, II, and III pneumoconiosis, which was consistent with the research results of Li et al. (19). This may indicate that patients with insurance could receive a better financial support and more actively go to hospitals for diagnosis or treatment to delay the progression of diseases. This study also found that there was no difference in the basic medical insurance coverage among patients in different stages. Meanwhile, relevant research showed that the concurrent rate of pneumoconiosis complications was lower in patients with employment injury insurance than in those without. This may indicate that compared with basic medical insurance, employment injury insurance and employer compensation were more important economic support for patients with pneumoconiosis. Wang et al. (20) found that poor economic status was an important influencing factor leading to pneumoconiosis patients not seeking medical services. Therefore, it is necessary to improve insurance coverage and strengthen the economic support for patients.

Related research (21, 22) showed that the life expectancy in Zhejiang Province in 1998, 2003, 2008, 2010, 2013, and 2015 were 73.9, 75.9, 76.7, 77.3, 77.8, and 77.7 years, respectively, and the average age at death in pneumoconiosis patients in the corresponding years were 65.2, 61.0, 66.3, 68.4, 70.3, and 70.4 years, respectively. The survival time in patients with pneumoconiosis was shorter than that in the general population. This study also found that with an increase in the stage of pneumoconiosis, the stage of first diagnosis, and the age at first diagnosis, the survival time in pneumoconiosis patients decreased, which agrees with the results of a study conducted in a coal mine in Jiangsu (23). A study found that the older the pneumoconiosis patients were, the higher the stage of pneumoconiosis was, and the more complications were developed in the pneumoconiosis patients, and eventually the survival time in patients was affected (24). It can be concluded that as the disease progresses, the damage caused by pneumoconiosis to health becomes increasingly serious. Therefore, it is necessary to conduct timely intervention and treatment for patients with pneumoconiosis to delay the disease progression and prevent potential infections and complications.

Currently, there has been no survival analysis in pneumoconiosis patients conducted in Zhejiang Province, and this study filled the gap in related knowledge. Based on the discussion of epidemiological characteristics, this study adopted the Kaplan-Meier method and Cox proportional risk regression model to conduct survival analysis. Compared with the analysis of epidemiological characteristics that can only observe the distribution characteristics of patients, survival analysis can explore the risk factors affecting the survival time in patients. The limitation of this study is that it is a retrospective follow-up survey, which may lead to inaccurate data for certain patients. In addition, this study does not investigate the factors that may affect the survival in patients, such as the type of work, enterprise type, enterprise scale, cigarette smoking, regular exercise, and other respiratory toxins.

## Conclusion

In summary, 13,812 pneumoconiosis cases were reported in Zhejiang Province from 1987 to 2019. Some patients had been exposed to dust for <5.0 years, suggesting that there were short-term dust-exposed patients in Zhejiang Province. Therefore, the occupational health management of key industries and working types should be strengthened. Industries with a high incidence of pneumoconiosis, such as mining, construction, and other industries should be supervised. In addition, providing financial support for patients and promoting further improvement of occupational disease insurance are also necessary to improve patient survival and quality of life. Patients with higher stage of pneumoconiosis at first diagnosis, older age, and longer duration of dust exposure had a higher hazard ratio of death.

## Data availability statement

The raw data supporting the conclusions of this article will be made available by the authors, without undue reservation. Requests to access the datasets should be directed to hzou@cdc.zj.cn.

## Ethics statement

The studies involving human participants were reviewed and approved by Ethics Committee of Zhejiang Provincial Center for Disease Control and Prevention. The patients/participants provided their written informed consent to participate in this study.

## Author contributions

HZ: investigation, formal analysis, and writing—original draft. ZS: formal analysis and visualization. YixZ: investigation and data curation. JZ: investigation. XF and YijZ: methodology and investigation. YH: formal analysis. XL: conceptualization and funding acquisition. LZ: writing—review and editing and supervision. All authors contributed to the article and approved the submitted version.

## Funding

This research was funded by the Health Commission of Zhejiang Province (Grant Numbers: 2019KY056 and 2019KY057).

## Conflict of interest

The authors declare that the research was conducted in the absence of any commercial or financial relationships that could be construed as a potential conflict of interest.

## Publisher's note

All claims expressed in this article are solely those of the authors and do not necessarily represent those of their affiliated organizations, or those of the publisher, the editors and the reviewers. Any product that may be evaluated in this article, or claim that may be made by its manufacturer, is not guaranteed or endorsed by the publisher.
